# A Numerical and Experimental Study on the Enrichment Performance of a Novel Multi-Physics Coupling Microchannel

**DOI:** 10.3390/mi16101146

**Published:** 2025-10-10

**Authors:** Qiao Liu, Ruiju Shi, Tongxu Gu

**Affiliations:** 1 Jihua Laboratory, Foshan 528000, China; 2 Suzhou Institute of Biomedical Engineering and Technology, Chinese Academy of Sciences, Suzhou 215163, China

**Keywords:** circulating tumor cells, separation, multi-field coupled simulation

## Abstract

The coupled method of inertial focusing and magnetic separation is effective for detecting and isolating circulating tumor cells (CTCs) from blood, wherein the design of a multi-physics coupled microfluidic device plays a critical role in the sorting efficiency. This paper presents a novel compact microfluidic device that combines inertial and magnetic forces for CTC separation. Using the finite element method, the effects of three major parameters (e.g., fluid velocity, particle properties, and magnetic field distribution) on sorting efficiency were comprehensively investigated and discussed. Simulated and experimental results demonstrate that the designed compact microfluidic device with coupled physical fields achieves high separation purity (>98%) for CTCs larger than 19 μm in diameter over a wide range of parameters, such as a fluid velocity greater than 3.5 × 10^−8^ m^3^/s, a remanent flux density between 1.08 T and 1.28 T, and the position of the magnet ranging from 2.5 mm to 4 mm.

## 1. Introduction

Cancer is a leading cause of death worldwide. In 2024, more than 200,000 new cancer cases and 61,000 cancer deaths were projected to occur in the United States [1]. It is believed that the occurrence of cancer metastasis is responsible for ~90% of cancer-related deaths [2]. However, many cancers (e.g., thyroid cancer and cervical cancer) are highly curable if diagnosed early and treated appropriately before metastasis develops [3,4]. CTCs, shed from primary tumors into the peripheral blood circulation, are the most important mediums of cancer metastases. Therefore, the identification and isolation of CTCs as a real-time liquid biopsy would contribute to the early detection of cancer and further advance the understanding of the metastatic cascade, tumor evolution, heterogeneity, and resistance to therapy [5,6,7,8]. Owing to the rarity of CTCs (1–100 CTCs per 1 mL of peripheral blood), conventional methods used for cell separation (e.g., centrifugation, chromatography, fluorescence, and magnetic-activated cell sorting) are either low-purity, inefficient, or expensive, and require highly skilled personnel [9]. Thus, it is necessary to propose more precise and efficient methods with operational capacity. In the 1990s, the concept of a micro-total analysis system was proposed and gradually developed into a microfluidic technology for controlling fluids in micro–nano-scale structures [10,11]. Due to the advantages of integration, automation, and high throughput, microfluidic technology has shown considerable promise in the isolation, enrichment, and purification of cells. And the advancement of microfluidics technology has facilitated the rapid development of various devices to improve the separation efficiency and recovery rate of the CTC isolation process. Those separation methods using microfluidics technology can be divided into two main kinds, namely active separation and passive separation. The former employs an external field such as dielectrophoresis, magnetophoresis, acoustophoresis, and optical tweezers to control cells with different biochemical or physical properties [12,13,14,15]. Meanwhile, passive separation (e.g., microfiltration, deterministic lateral displacement, pinch flow fractionation, and inertial focusing) only depends on the microstructure of the channel and the fluid dynamics [16]. As one of the most powerful and accurate passive separation methods, inertial focusing has been researched and developed extensively due to its ideal operational capacity, high throughput, and high collection rate [17,18]. Inertial focusing is the phenomenon where particles suspended in a fluid stream may move across the flow lines and fall into stable positions at certain cross-sectional regions, and the equilibrium position of suspended particles is affected by the wall effect, Reynolds number, their characteristics (e.g., deformability, initial shape, size, density ratio), viscosity ratio, ambient fluid, etc. [19]. Accordingly, inertial focusing devices used for the separation of CTCs have been increasingly proposed in recent years. One of the most widely used geometries for separating cells is a spiral channel, in which the number of equilibrium positions are reduced to one position owing to the Dean flow [2,20,21,22,23]. Compared with traditional spiral channels with rectangular cross sections, increasing heterotypic microchannels with spiral–contraction–expansion [24], multistage loops [25], and U-shaped and W-shaped cross-sections [26] have been designed for highly efficient separation of the cell or other microbes. Admittedly, these devices adopting spiral channels have achieved high cell separation efficiency (>80%). However, their recovery rate or purity is usually low due to the selectivity only based on size, which impedes the transformation of experimental achievement into commercial clinical applications. Thus, other separation methods based on biological properties were applied as external supplement for further purification, especially dielectrophoresis [27] and magnetophoresis [28]. On the one hand, the method of coupling inertial focusing and other technologies usually involves various physical fields such as the fluid field, magnetic field, and electric field. Obviously, with the addition of the physical field, the design of separation devices becomes increasing complex, requiring researchers to master multidisciplinary knowledge. On the other hand, the multi-variable characteristics (e.g., microchannel structure, fluid velocity, particle dynamics size, and the magnitude of external force) pose realistic challenges to the design and fabrication of the inertial focusing system.

Recently, owing to advances in computer technology, numerical modeling has become a crucial tool for predicting and assessing the underlying physics and performance of inertial microfluidic devices, ultimately facilitating their optimum design for a high throughput, recovery rate, and purity. For instance, Afshin et al. performed three-dimensional simulations to demonstrate the separation efficiency of a single-loop spiral microchannel [2]. Hyungkook et al. [29] analyzed channel deformation in two polydimethylsiloxane (PDMS) spiral devices using numerical simulation and confocal imaging. Based on this deformation analysis, they optimized PDMS devices for blood separation and Chinese hamster ovary (CHO) cell retention and translated the designs into plastic versions by compensating for the channel deformation. Zhao et al. [30] developed a dimension-confined spiral microchannel with an ultra-low aspect ratio to achieve flow rate and particle size insensitive inertial focusing. The equally distributing microstructures were designed in the spiral microchannel to enhance secondary flow, which was explored by computational fluid dynamics simulation. The numerical simulations mentioned above, which were used to evaluate the feasibility of inertial microfluidic designs, primarily aimed to reveal the focusing behavior of specific particles under specific conditions, thereby validating the effectiveness of the channel structure and Reynolds number (Re) setting. However, to meet the design requirements of inertial microfluidic systems for efficient separation of various types of actual particles, inertial microfluidics have been integrated with active separation methods, and the diversity and complexity of microfluidic structures have continued to increase accordingly. Consequently, numerical studies focusing solely on inertial effects are often insufficient. There is a pressing need to establish multi-physics coupled numerical models capable of accurately predicting particle focusing positions under the influence of multiple physical fields.

In this study, a multi-field coupled numerical study is performed on the inertial and magnetic separation of various CTCs from red and white blood cells (RBCs and WBCs) using an irregular spiral microchannel and an added magnetic field induced by a sector magnet. In this system, a blood sample containing CTCs is injected into the microchannel. The cells are then separated into distinct streams based on their different equilibrium positions, which are determined by the combined effects of inertial focusing and the magnetic field, and are subsequently collected through two separate outlets. This study comprehensively investigates key parameters affecting separation efficiency, including fluid velocity, particle properties (e.g., size and magnetic susceptibility), and magnetic field strength. Furthermore, the optimal set of parameters for achieving high-purity CTC separation is identified.

## 2. Theory and Mathematical Formulation

### 2.1. Principle of Operation

In the 1960s, Segre et al. observed that particles formed an annulus in a straight pipe owing to inertial forces. The inertial lift forces (*F_L_*), induced by shear gradient and wall effects, were found to be related to fluid density, flow rate, particle diameter, and channel cross section. In Poiseuille flow, the net force acting on particles whose size is comparable to microchannel dimension *a*_c_/*h* > 0.07 is as follows [31]:

(1) FL=ρUm2a4Dh2fL(Rec,xc)

where *ρ*, *U*_m_, and *a* are the fluid density, particle diameter, and maximum fluid velocity, respectively. And *f*_L_ is the lift coefficient, which is a function of the position of the particle (*x*_c_) and channel Reynolds number (Re). *D*_h_ is the hydraulic diameter related to the channel cross section, which can be expressed as $D_{h}=2wh/(w+h)$ for a rectangular channel.

Compared to straight channels, curved channels are dimensionally compact, and have been widely used for the rapid and continuous isolation of viable CTCs. Di Carlo et al. [32,33] demonstrated that the introduction of curvature introduces a secondary cross-sectional flow field perpendicular to the primary flow direction, known as Dean flow. The magnitude of Dean flow is measured using a nondimensional parameter, the Dean number (De) [32]:

(2) De=ρUDhμDh2RC=ReDh2RC

where *U*, *μ*, and *R*_C_ are the average flow velocity, dynamic viscosity, and radius of curvature of the path of the channel, respectively. A drag force generated by Dean flow, namely Dean drag force (*F*_D_), entrains and drives particles along the vortex flow direction. *F*_D_ is related to fluid density, particle size, and Dean number, and its magnitude can be calculated by Stokes’ law (Equation (2)) [31].

(3) $$F_{D}=3\pi \mu U_{\mathrm{Dean}}a_{c}$$

where *U*_Dean_ is the lateral migration velocity of particles flowing in a channel, named Dean velocity. It depends on the Dean number and can be calculated as follows:

(4) $$U_{\mathrm{Dean}}=1.8\times 10^{-4}De^{1.63}$$

**Figure 1:**
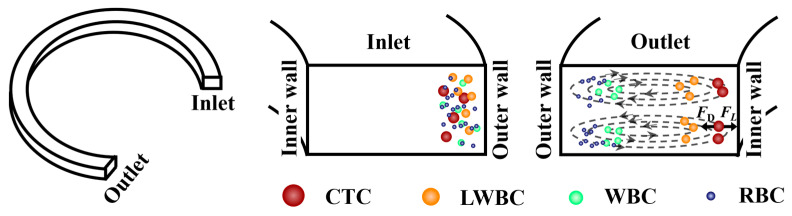
The equilibrium positions of particles migrating in microchannels with curvilinear geometry.

The cells or particles experience both *F*_L_ and *F*_D_ when migrating in microchannels with curvilinear geometry. The interplay between these forces reduces the equilibrium positions to two near the inner channel wall, located within the upper and lower Dean vortices, as clearly shown in Figure 1.

### 2.2. Principle of Simulation Settings

#### 2.2.1. Design of Biochip

**Figure 2:**
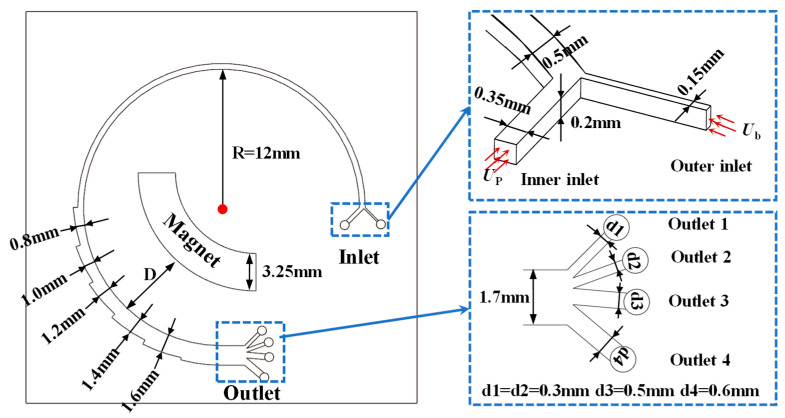
A schematic of the research microchip.

In this study, the separation of CTCs from blood cells is investigated using a novel inertial spiral microchannel coupled with a magnetic field. A schematic of the research microchip is shown in Figure 2. The proposed microchannel consists of two parts, a curved spiral microchannel with a constant width and a curved spiral microchannel with increasing width near the superparamagnetic magnet. The former is used to separate cells with different sizes, and the latter is supplemented to separate cells with similar sizes based on biochemical properties. The incremental size in channel width depends on the balance of the fluidic speed of magnetic separation techniques in microfluidic separation devices and the processing size limit of 3D printing. It has been set as 0.2 mm to obtain a congenial fluidic speed for effective magnetic separation. The curved spiral microchannel part contains two inlets and four outlets. Bloodstream containing CTCs is injected into the microchannel using a pressure controller from the outer inlet. And the inner inlet is used for the injection of Phosphate-Buffered Saline (PBS) to create a tight flow in the vicinity of the outer channel wall. The fundamental microstructures of the curved spiral microchannel with dimensions are illustrated in Figure 2. To comprehensively study the effects of parameters on the performance of the chip, a series of parameters are designed in this article, as shown in Table 1, including the fluid velocity, magnetic field, and particle properties. First, the PBS fluid velocity (Up) is the main parameter affecting the separation of the cells, which is set from 2.5 × 10^−8^ m^3^/s to 3.5 × 10^−8^ m^3^/s. Second, the magnetic field induced by the superparamagnetic magnet also affects the separation performance for similar cells or particles, which is varied by altering the residual magnetic flux density and the distance between the channel and the magnet. Finally, the separation efficiency of the spiral microchannel for CTCs with different particle size is also examined.

#### 2.2.2. Design of Simulation Settings

**Figure 3:**
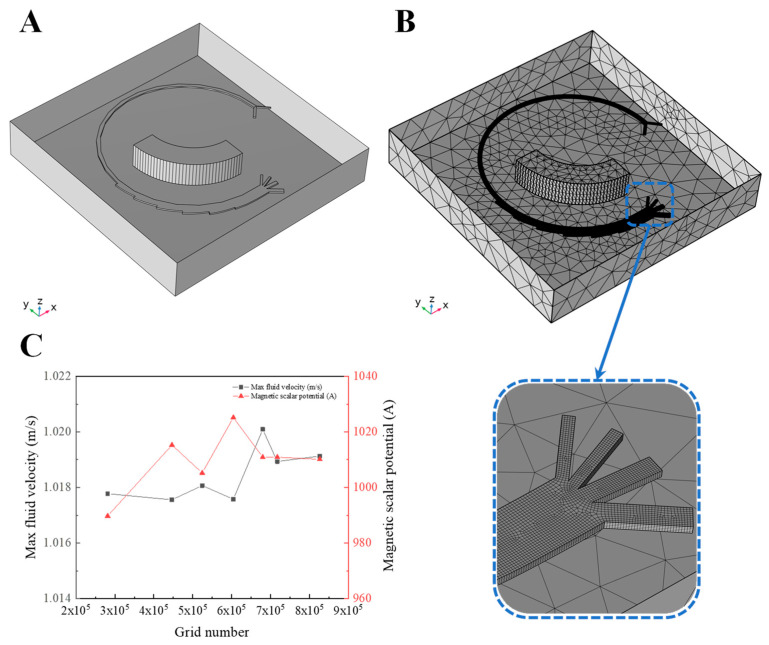
(**A**) The simplified microchannel and a schematic of the research microchip pre-processing stage. (**B**) The grid setting of different channel structures. (**C**) A plot of the velocity and magnetic scalar potential with the increment in grid number to prove mesh convergence.

With the development of computer technology, commercial CFD packages have been widely used by investigators, e.g., ANSYS Fluent, COMSOL Multiphysics and OpenFOAM. Among these, COMSOL Multiphysics 6.1 was selected for this study because it provides the necessary modules (e.g., Particle Tracing and Magnetic Fields) and offers excellent multi-field coupling capabilities. A standard numerical study typically involves three procedures: pre-processing, calculation, and post-processing. To ensure smooth simulations and accurate results, it is essential to conduct reasonable pre-processing. In this work, the pre-processing stage consists of five steps: geometry, material, physics field, mesh, and study setup. The geometry is simplified in Figure 3A, which preserves the main microstructure (e.g., microchannel and magnet) and ignores other redundant chip structures without exerting negative effects on the simulation results. The material setting is clearly shown in Figure 3 considering the actual experiment, including the Newtonian fluid, plastic material, and magnet material. Three physics interfaces are then introduced: the Laminar Flow (SPF) interface, Magnetic Fields and No Currents (MFNC) interface, and the Particle Tracing for Fluid Flow (FPT) interface. In this step, specification of the appropriate boundary conditions is critical for obtaining reliable simulation results. The main parameters of the physics interfaces are therefore carefully defined, as summarized in Table 2. According to the parameters, the range of forces exerted on particles can be calculated or simulated as shown in Table 3. Then, the mesh is generated according to the region and physics properties, ensuring a balance between computational efficiency and simulation accuracy. The element size of the main microchannel for fluid flow is set extremely fine, while the other structures are meshed with a normal resolution, as shown in Figure 3B. And grid resolution with tetrahedral elements is determined based on calculating the maximum Dean velocity for various Reynolds numbers [2]. In this study, the grid independence and convergence have been proven through a series of simulations by gradually increasing the number of grids, as shown in Figure 3C. To obtain a stable flow field and magnetic field, the total number of elements is at least 670,000. Finally, the study is divided into two stages: a stationary study and a time-dependent study. The former is used to calculate MFNC and SPF to obtain the corresponding fluid field and magnetic field in a short time. The latter is used to calculate FPT to track the particle trajectory within the microchannel. The time step of the time-dependent study is set to 1 ms, and the final solution from the stationary study is used as the initial condition for the transient analysis.

## 3. Numerical Simulation and Results

### 3.1. Simulation of Fluid Flow in Magnetic Field

#### Flow Field Analysis

**Figure 4:**
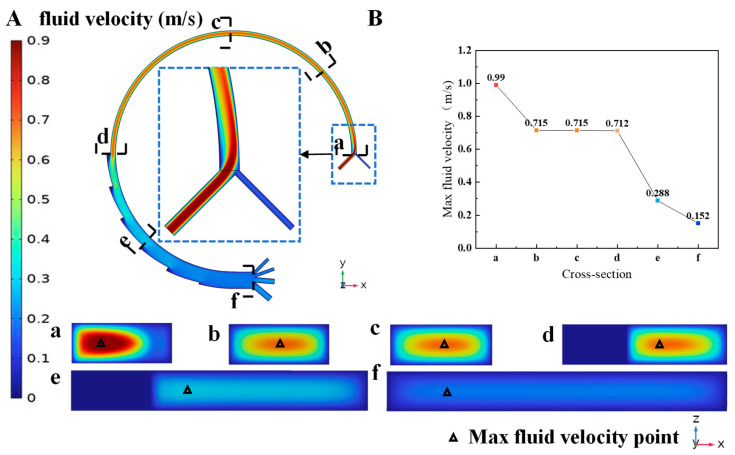
The simulation results of fluid flow. (**A**) The fluid flow velocity in the microchannel in the x–y direction cross section and the x–z direction cross section, the letters a–f respectively represent the cross-sectional velocity distribution cloud diagrams at different positions. (**B**) A plot of the max fluid velocity alteration in the channel.

After implementing the multi-field coupled model, the flow field is obtained by static analysis. The distribution of flow field can then be visualized through post-processing of the simulation results. Figure 4 illustrates the fluid velocity distribution in the microchannel of case T0. The blood with cells is injected into the microchannel through the outer inlet and then diluted by PBS solution. As the mixing process progresses, the mixed solution gradually exhibits Newtonian fluid properties. The flow velocity reaches its maximum at the channel center and decreases as the distance from the channel wall decreases. Specifically, the flow field in the spiral curved channel with an increasing cross section is more stable and uniform than that in the constant curved channel, which is beneficial for magnetic separation. To analyze fluid flow at the cross section of the channel perpendicular to fluid flow direction, five x–z direction cross sections are selected in this particle to show the distribution of velocity. The fluid velocity in cross section A is maximal and the high-velocity region is near the inner channel. As the fluid flows and mixes, the point of maximum fluid velocity migrates from the inner wall to the outer wall over a short distance, and then gradually shifts back from the outer wall to the inner wall. Meanwhile, the medium velocity region is enlarged and the value of max fluid velocity decreases significantly.

The flow field, which is influenced by channel structure and fluid velocity, affects the separation of cells. As the direct influencing factor of the flow field, the PBS velocity (U_p_) at the inner inlet is varied to generate different flow fields, while keeping the blood solution velocity (U_b_) constant. With increasing U_p_, the max fluid velocity is increased from 0.989 m/s to 1.134 m/s, while the transition region between high and low flow velocities is significantly reduced.

### 3.2. Particle Motion in Multi-Physics Coupling Field

**Figure 5:**
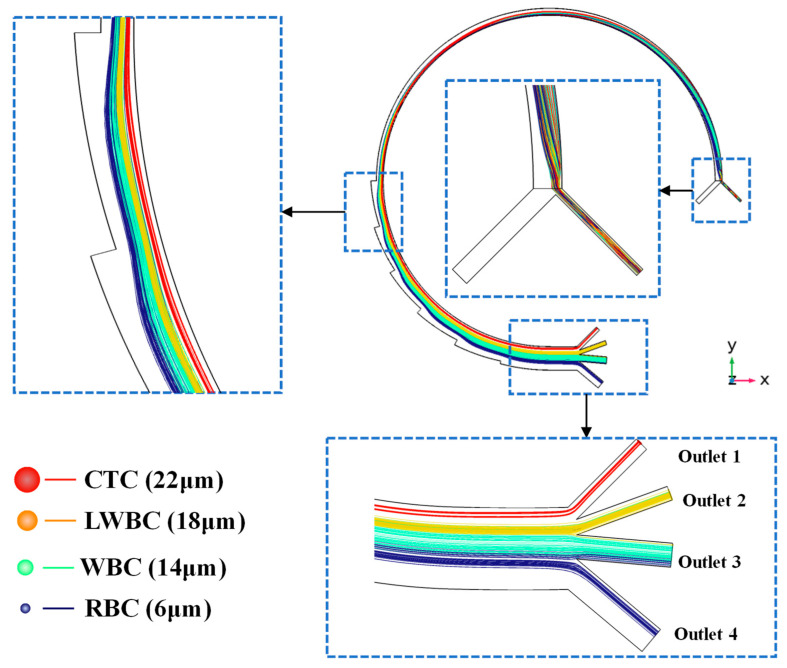
The simulation results of cell trajectories inside the microchannel at 600 ms.

According to the simulation results of the coupled fluid and magnetic field, the average fluid velocity is about 0.5 m/s. The simulation time of particle migration in the channel is set to 0.6 s based on channel length and the average fluid velocity. It takes about 230 ms for most cells to travel through the entire channel and reach the outlet, and the trajectories of cell motion from the inlet to the outlet are shown in Figure 5. This figure clearly shows the distribution of particles with different diameters within key channel structures. Cells travel through the short inlet channel into the main curved separation channel within 20 ms, and are then distributed near the outer wall due to the effect of the sheath flow. As times progresses, the cells migrate toward the inner wall along Dean vortices. When the CTCs and larger WBCs reach the position near the inner wall, the interplay of the inertial lift force and the drag force causes cells to accumulate at two equilibrium positions near the inner channel. Since CTCs are assumed to be magnetic, with a particle relative permeability of 1.08, they are attracted to the right side of the channel near the magnet by a magnetic force as the cells travel through the curved spiral microchannel. Finally, the CTCs and larger WBCs (LWBCs) are separated and exit from Outlet 1 and Outlet 2, respectively. The red lines represent the trajectories of CTC motion, which clearly indicate that almost all CTCs exit through Outlet 1 with high purity. As for the small cells, they continue to move along the Dean vortices and return to the position near the outer wall, and are then directed towards the outer side due to the diffusion flow caused by the expanded channel. Finally, the smaller WBCs and red blood cells exit through Outlet 3 and Outlet 4.

### 3.3. Analysis of Parameter Effects on Cell Motion

**Figure 6:**
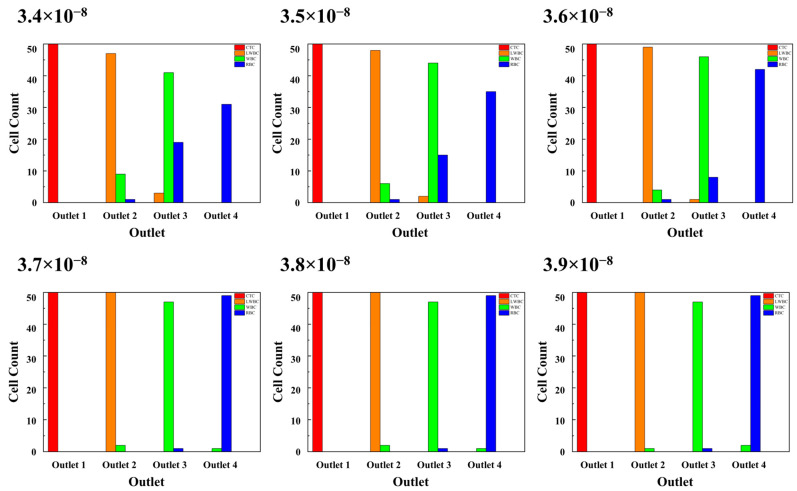
The cell counts at different outlets for various Up values.

**Figure 7:**
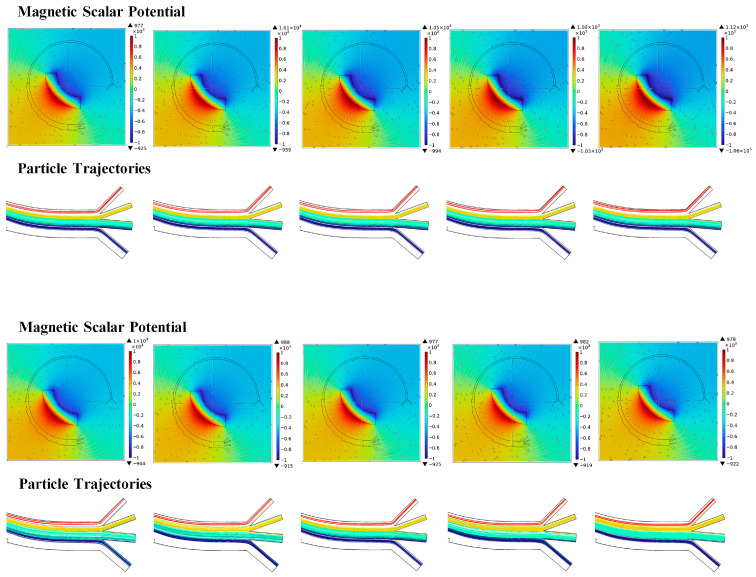
The cell trajectory at different outlets for various parameters.

In this study, three types of parameters are considered the main factors affecting cell separation, namely the fluid velocity, magnetic field, and particles properties. To evaluate the effects of these parameters, the numbers of cells collected at the four outlets are counted after simulation. Based on the data, a series of diagrams are drawn, as shown in Figure 6 and Figure 7.

#### 3.3.1. Fluid Velocity

The novel microchannel in this study has two inlets for the input of blood and PBS solution, and the interaction between the initial fluid velocity of both determines the average fluid velocity of the spiral channel. In this study, the initial U_b_ is a constant value (2.217 × 10^−9^ m^3^/s), while U_p_ has been changed from 3.4 × 10^−8^ m^3^/s to 3.9 × 10^−8^ m^3^/s with an increment of 0.1 m^3^/s. As shown in Figure 6, the number of CTCs reaching Outlet 1 is 50 under all simulation conditions with different U_p_ values. The separation efficiency and the purity of CTCs both reach 100%. However, the purity of the other cells at other outlets is relatively lower when U_p_ is below 3.7 × 10^−8^ m^3^/s. At a lower U_p_, the smaller cells tend to exit through the outlet designated for larger cells. In particular, almost half of the RBCs arrive at Outlet 3 when U_p_ is 3.4 × 10^−8^ m^3^/s. As U_p_ increases, the purity of cells in different outlets is increased and tends towards being stable when U_p_ exceeds 3.7 × 10^−8^ m^3^/s. The simulation results reveal that the final separation efficiencies of CTCs in the above cases are similarly high when using this novel microchannel. Moreover, the separation efficiencies of other cells can also be improved by adjusting U_p_ to an appropriate value from the lower value.

#### 3.3.2. Magnetic Field

The last factor is the magnetic field, which depends on the remanent flux density of the magnet and its position in the microfluidic chip. In this study, the remanent flux density is set in the range of 1.08–1.28 T, based on the properties of the commonly used magnet. As seen in Figure 7, the magnetic scalar potential is significant increased with increasing remanent flux density. With the increasing magnetic force, the CTCs, which are assumed to possess magnetic properties, are driven to a position closer to the magnet in Outlet 1. A portion of CTCs tend to adhere on the inner wall of Outlet 1 when the magnetic scalar potential reaches 1.12 × 10^3^ owing to the increase in magnetic force. The settled positions of the magnet are chosen on the inside of the microfluidic chip, and the linear distance from the center (Dc) is selected as 2.5–4 mm to induce various magnetic fields. As seen in Figure 7, the range and value of the magnetic scalar potential both vary when reducing the distance between the magnet and the microchannel. Compared with the remanent flux density, the effect of magnet position on CTC sorting is more complex. Adjusting the position of the magnet not only changes the outflow position of CTCs in Outlet 1, but also significantly affects the distribution of other cells at different outlets. As Dc increases, a greater number of RBCs tend to exit through Outlet 1, and the separation purity of cells at each outlet is enhanced.

#### 3.3.3. Particle Properties

**Figure 8:**
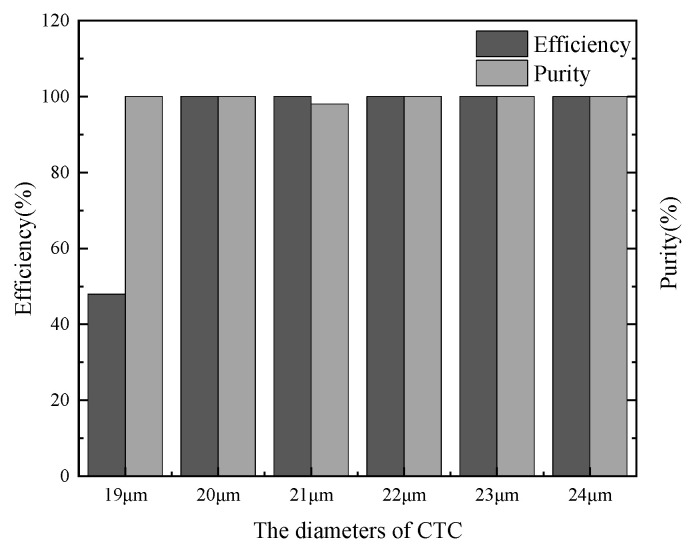
The efficiency and purity of various simulation tests for different CTCs with various diameters.

As another factor, particle size is also investigated numerically due to the non-uniformity of CTCs across different cancer patients and their variability even in the same patient. The diameters of various CTCs range from 16 to 24 μm, and they are assumed to be 19–24 μm when other blood cells are assumed as 6 μm, 14 μm, and 18 μm in this study. The proposed standard microchannel is used to separate CTCs with five different diameters, and the efficiency and purity of CTC collection at Outlet 1 are clearly shown in Figure 8. Beside the small CTCs with a diameter of 19 μm, almost all CTCs exit through this outlet with high purity. However, owing to the interaction between the fluid field and the magnetic field, a portion of larger CTCs (Dp ≥ 23 μm) tend to adhere on the inner wall of Outlet 1. This phenomenon occurs because larger CTCs experience stronger inertial and magnetic forces when traveling through the channel. Those CTCs adhering on the wall can be released by removing the moveable magnet after the entire blood sample has been processed.

## 4. Experiment and Results

**Figure 9:**
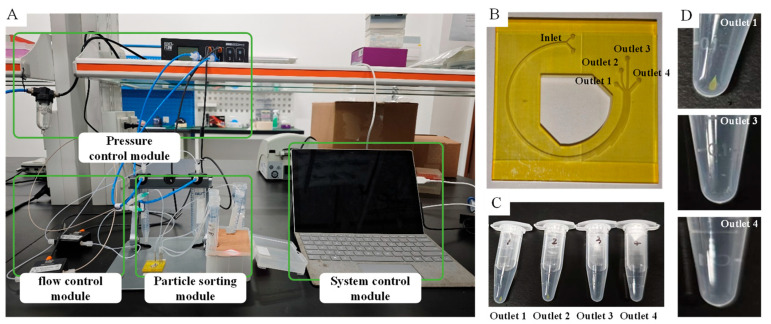
The separating system and experiment of microbeads with different diameters. (**A**) The separating system for conducting the sorting experiment. (**B**) A photograph of the ideal microfluidic chip. (**C**) The recovered solution from the outlets of the microfluidic chip. (**D**) An amplified picture of the collecting tube used to recover the solution.

To evaluate the availability of this microfluidic chip for clinical application, the experiments have been carried out based on a separating system. This system consists of four significant modules (e.g., pressure control module, flow control module, particle sorting module, and system control module), as shown in Figure 9. The crucial equipment of the particle sorting module is an ideal microfluidic chip fabricated by precise 3D printing technology whose machining accuracy is better than ±25 microns. Four distinct microspheres with different diameters and fluorescence characteristics have been used in separating experiments. They are red polystyrene microspheres with a diameter of 5 µm, blue polystyrene microspheres with a diameter of 14 µm, green polystyrene microspheres with a diameter of 20 µm, and yellow fluorescent carboxyl magnetic microspheres with a diameter in the range of 18–24.9 µm. The last one is assumed as CTCs with magnetization treatment when other microspheres are assumed as blood cells.

Before the experiments, the pressure control module and flow control module were precisely calibrated. The initial U_b_ was set as 2.217 × 10^−9^ m^3^/s according to the parameter of the numerical simulation, while U_p_ was set as 3.5 × 10^−8^ m^3^/s. The mixture of four microspheres was diluted in 665 µL physiological saline and was completely separated by the particle sorting module within 5 min after the system was switched on. The recovered solutions from the outlets of the microfluidic chip need to be enriched before fluorescent recognition, and the concentrated solution collected from Outlet 1 contains a significant number of yellow fluorescent carboxyl magnetic microspheres, as shown in Figure 9C.

**Figure 10:**
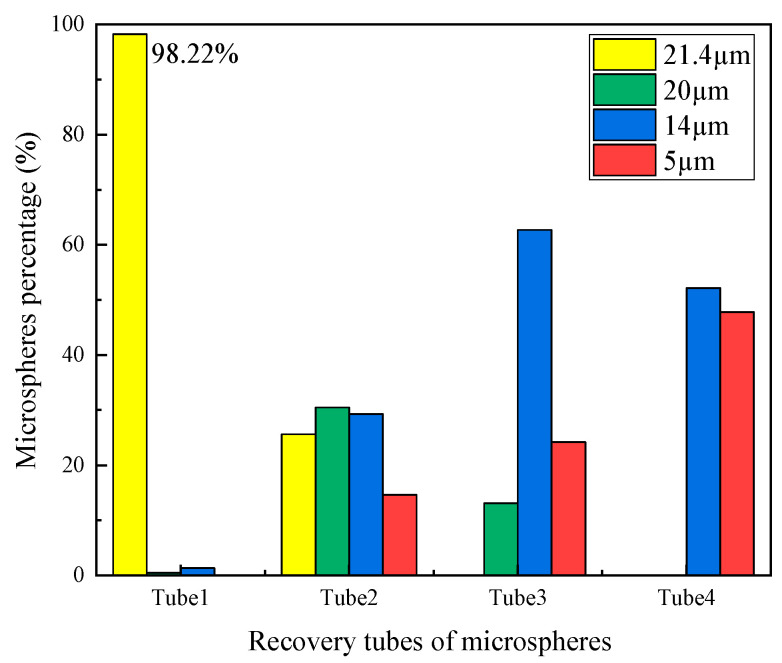
The percentage of various microspheres in different recovery tubes by fluorescence determination.

To evaluate the results of the separating experiments using the proposed microfluidic chip, the microspheres dispersed in concentrated solution were detected and counted by using an advanced flow cytometer from Beckman. The results confirmed the effective separation of CTCs using the proposed microfluidic chip with a curved spiral microchannel, which is consistent with the simulation results. As shown in Figure 10, almost all of the yellow fluorescent carboxyl magnetic microspheres, which represent CTCs, migrate toward the inner wall due to the interplay of the inertial lift force and the drag force, and then flow in tube 1 from Outlet 1. The purity of yellow fluorescent carboxyl magnetic microspheres in tube 1 exceeds 98%. Meanwhile, other microspheres have been recovered in tubes 2–4. The identification results of the microspheres from fluorometric measurements demonstrate the effectiveness of the proposed microfluidic chip with a curved spiral microchannel. The chip is characterized by excellent operational efficiency and simplicity, and could provide a novel approach for clinical applications.

## 5. Conclusions and Discussion

In contrast to cascaded microfluidic chips, where the effects of the fluid field and the magnetic field are relatively independent, the novel chip presented in this study couples inertial focusing with magnetic separation in a compact structure. By establishing a numerical model of the coupled physical fields, the influence of three critical parameters (e.g., fluid velocity, remanent flux density, and the position of magnet) on the sorting efficiency of CTCs from RBCs and WBCs in the spiral microchannel was investigated. The results indicate that a higher fluid velocity enhances inertial focusing, leading to improved sorting performance. Similarly, an increase in the remanent flux density and a reduction in the position of the magnet favor the separation of CTCs. However, when the remanent flux density exceeds a certain threshold or the position of the magnet falls below a certain threshold, CTCs tend to adhere to the inner wall of the channel, which adversely affects the separation efficiency. The device achieved a separation purity exceeding 98% for CTCs represented by magnetic microspheres with diameters of 20–24 μm under various conditions, specifically fluid velocity exceeding 3.5 × 10^−8^ m^3^/s, remanent flux density between 1.08 T and 1.28 T, and the position of the magnet ranging from 2.5 mm to 4 mm. Furthermore, using this coupled chip, other microspheres (e.g., LWBCs, WBCs, and RBCs) can also be recovered via different outlets for subsequent downstream analysis. In summary, the multi-physics coupled numerical model developed in this study provides a valuable tool for designing ideal microfluidic devices for CTC separation. The proposed device architecture is readily manufacturable for practical applications.

## Figures and Tables

**Table 1:** The series of parameters designed in this article.

Parameters		Value	Unit
Fluid velocity	Up	3.4 × 10^−8^, 3.5 × 10^−8^, 3.6 × 10^−8^, 3.7 × 10^−8^, 3.8 × 10^−8^, 3.9 × 10^−8^	m^3^/s
Magnetic field	D	2.50, 2.75, 3.00, 3.25, 3.50, 3.75	mm
	Remanent flux density	1.08, 1.12, 1.16, 1.20, 1.24, 1.28	T
Particle properties	d_p_ (CTC)	19, 20, 21, 22, 23, 24	μm

**Table 2:** The main parameters and settings of the physics interfaces.

Physics Interfaces	Major Parameters		Value	Unit
SPF	Fluid properties	Default discretization	P2 + P2	-
		Density	1000	kg/m^3^
		Dynamic viscosity	1 × 10^−3^	Pa × s
	Flow channel parameters	Hydraulic diameter	2.86 × 10^−4^	m
		Reynolds number	40–200	-
		Dean number	5–25	-
	Boundary condition	wall	No slip	-
		Inner inlet flow rate	3.4 × 10^−8^–3.9 × 10^−8^	m^3^/s
		Outer inlet flow rate	2.217 × 10^−9^	m^3^/s
		Outlet pressure	0	N
MFNC	Relative permittivity		1	1
	Remanent flux density		1.08–1.28	T
	Position of magnet		2.5–3.75	mm
	Magnetic flux density		B = μ_0_μ_r_H + B_r_	T
FPT	Wall conditions		Adhesion	
	Particle properties	Density	1050	kg/m^3^
		Diameter	6, 14, 18, 19/20/21/22/23/24	μm
	Particle number		50	-
	Drag force		From SPF of simulation	-
	Lift force		From SPF of simulation	-
	Magnetophoretic force	Particle relative permeability	1.08	-

**Table 3:** The numerical range of forces calculated under the main parameters.

**Forces**	Parameters/Formula		Value	Unit
F_D_	Diameter	6 μm	1.5 × 10^−10^–3.18 × 10^−9^	N
		24 μm	6.0 × 10^−9^–1.27 × 10^−8^	N
F_L_	Diameter	6 μm	7.39 × 10^−13^–3.13 × 10^−11^	N
		24 μm	1.89 × 10^−10^–8.02 × 10^−9^	N
Magnetic force ($F_{map}$)	$F_{map}=2\pi r_{p}^{3}\mu_{0}\mu_{r}K\nabla H^{2}$		From FPT of simulation	N

## Data Availability

The original contributions presented in this study are included in the article. Further inquiries can be directed to the corresponding author.
